# Discovery of an Octahedral Silicon Complex as a Potent Antifungal Agent

**DOI:** 10.3390/molecules22040637

**Published:** 2017-04-15

**Authors:** Chen Fu, Bin Fu, Xixi Peng, Guojian Liao

**Affiliations:** College of Pharmaceutical Sciences, Southwest University, Chongqing 400715, China; fubin3399@email.swu.edu.cn (B.F.); xxi151515@163.com (X.P.)

**Keywords:** silicon, octahedral complex, antimicrobial activity, antifungal agent

## Abstract

Octahedral transition metal complexes have been shown to have tremendous applications in chemical biology and medicinal chemistry. Meanwhile, structural transition metals can be replaced by inert octahedral silicon in a proof-of-principle study. We here introduce the first example of octahedral silicon complexes, which can very well serve as an efficient antimicrobial agent. The typical silicon arenediolate complex **1** {[(phen)_2_Si(OO)](PF_6_)_2_, with phen = 1,10-phenanthroline, OO = 9,10-phenanthrenediolate} exhibited significant inhibition towards the growth of *Cryptococcus neoformans* with MIC and MFC values of 4.5 and 11.3 μM, respectively. Moreover, it was fungicidal against both proliferative and quiescent Cryptococcus cells. This work may set the stage for the development of novel antifungal drugs based upon hexacoodinate silicon scaffolds.

## 1. Introduction

Cryptococcosis is an invasive mycosis associated with significant morbidity and mortality [1]. Cryptococcal meningitis, caused by *Cryptococcus neoformans*, leads to approximately 600,000 deaths from 1,000,000 infections annually [2], which is even exacerbated by the emergence of drug resistance [3,4,5,6]. Currently, an acute cryptococcal infection is treated with amphotericin B or azole in combination with 5-flucytosine [7]. However, due to the renal toxicity and lack of oral formulations of amphotericin B, hematological toxicity of 5-flucytosine [8], and the surge of drug resistance, there is an urgent need for the development of new antimicrobials. Exploring novel antifungal agents with new mechanism or chemical structure is intensely desired [9,10].

Several years ago, one of us reported the first examples of designed biologically active complexes based on octahedral silicon and found that silicon complexes can be viewed as template-substituent of ruthenium complexes [11,12,13]. As a structural octahedral center, the metalloid silicon owns distinct advantages over transition metals, such as a lack of toxicity concerns and high stability in configuration [14,15]. Similarly, as the octahedral polypyridyl ruthenium complexes, hexacoordinate silicon arenediloate complexes could intercalate and remarkably stabilize DNA duplex, which means enormous biological application potentiality [16,17,18,19,20].

## 2. Results and Discussion

### 2.1. Antimicrobial Activity

**Figure 1:**
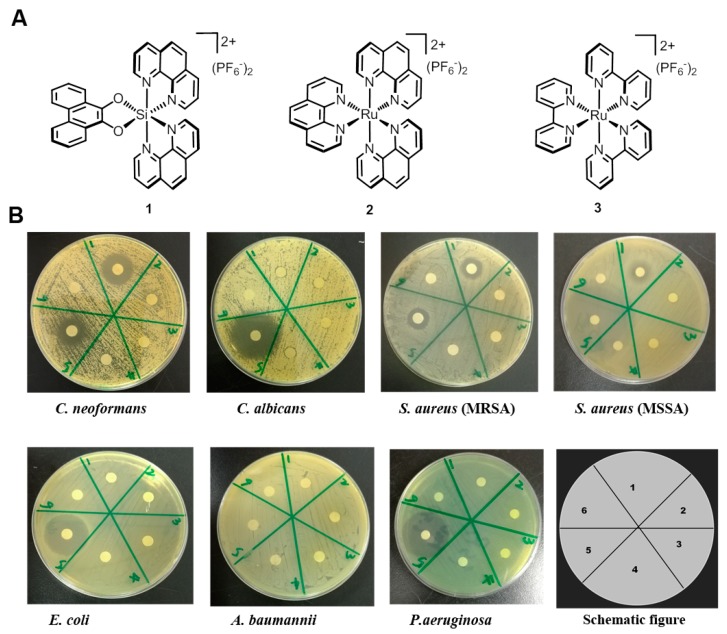
Anti-bacterial and anti-fungal activity of Complex **1** as determined by the disk diffusion assay. (**A**) Chemical structures of Complexes **1**, **2**, and **3**. (**B**) Strains including *C. neoformans* H99, *C. albicans* ATCC90028, *S. aureus* ATCC 33591(MRSA), ATCC 25923(MSSA), *E. coli* ATCC25922, *P. aeruginosa* PAO1, and *A. baumannii*, a clinical strain resistant towards *β*-lactam antibiotics. The corresponding substances in the schematic figure: Complex **1** (1), Complex **2** (2), Complex **3** (3), DMSO (4), or water (6) was used as negative control; ampicillin (5) was used as a positive control for antibacterial agents and fluconazole (5) as a positive control for antifungal agents, respectively.

Firstly, we carefully studied antimicrobial activities of the typical octahedral silicon complex **1**, and two ruthenium complexes—**2** {[(phen)_3_Ru](PF_6_)_2_, with phen = 1,10-phenanthroline} and **3** {[(bpy)_3_Ru](PF_6_)_2_, with bpy = 2,2′-bipyridine}—with an analogous structure as control compounds (Figure 1). The antibacterial and antifungal activities of the complexes **1**–**3** were firstly investigated with Kirby–Bauer disc diffusion assays [21]. In this assay, five different bacterial strains (*Staphylococcus aureus* MRSA and MSSA, *Escherichia coli*, *Pseudomona saeruginosa* PAO1, and *Acetobacter baumannii*) and two fungal strains (*Candida albicans* and *Cryptococcus neoformans*) were plated on an agar dish, and disks soaked with solution of the complexes **1**–**3** (25 μg) or with ampicillin (25 μg) as the positive control for bacteria or fluconazole (25 μg) as the positive control for fungi. After 24 h for bacterial strains, or 48 h for fungal strains, the growth inhibition zones of the test strains were determined. As shown in Figure 1, the ruthenium complexes **2** and **3** did not show any obvious antimicrobial activity. In contrast, the silicon complex **1** displayed potent anti-bacterial activity towards *S. aureus*, including the MRSA, whereas it was inactive to Gram-negative bacteria (*E. coli*, *P. aeruginosa*, and *A. baumannii*). More interestingly, Complex **1** exhibited selective anti-fungal activity towards *C. neoformans*, one major opportunistic fungal pathogen, which could cause systemic infections that are commonly involved in the central nervous system (CNS).

### 2.2. Determination of MIC Values

Based on the selective anti-fungal activity that Complex **1** exhibited, we further determined the minimum inhibitory concentration (MIC) value of Complex **1** against *C. neoformans*. Accordingly, serial solutions of Complex **1** prepared in DMSO with concentrations ranging from 0.56 to 71.68 μM were added to mid-log phase of *C. neoformans* and incubated 2 days to observe the cell growth. The MIC was determined as the lowest concentration of Complex **1** inhibiting visible fungal growth. Meanwhile, we also set the ruthenium complexes **2** and **3** and typical positive drugs (fluconazole, amphotericin B, and 5-flucytosine) as the control group and determined the MIC values as well. As displayed in Table 1, it is not surprising that amphotericin B inhibited the best antifungal activity against the growth of *C. neoformans* with an MIC value of 0.3 μM. However, the results showed that the MIC value of Complex **1** was 4.5 μM, which exhibited better antifungal activity than fluconazole (6.5 μM) and was comparable to 5-flucytosine (3.9 μM). In contrast, the ruthenium complexes **2** and **3** showed only very limited activity against *C. neoformans*, with MIC values of 34.3 μM and 74.4 μM, respectively. We are convinced that the silicon complex could exhibit better antifungal activity with proper modification in the future.

### 2.3. Determination of MFC Value of Complex ***1***

**Figure 2:**
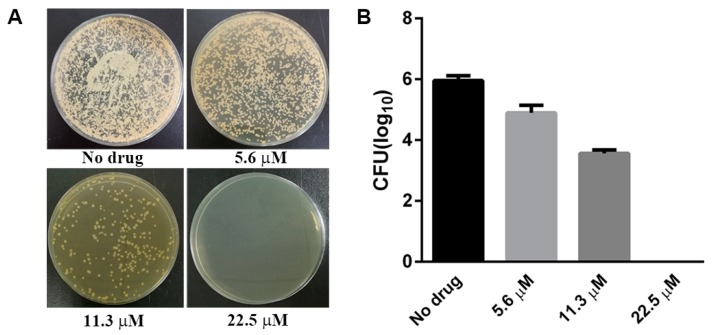
Inhibition of the growth of *C. neoformans* by Complex **1** as determined by the broth dilution method. (**A**) Qualitative analysis of colonies at the different concentrations of Complex **1**; (**B**) Quantitative analysis of colony-forming units (CFUs) at the different concentrations of Complex **1**. Results are representative of three independent experiments. CFUs were determined at 48 h after incubation and are reported as an average ± S.D.

Considering the selective anti-fungal activity towards *C. neoformans* was our capital concern, we only focused on the cryptococcal relevant work of Complex **1** in the following study. We next determined the minimum fungicidal concentration (MFC) of Complex **1** following the agar dilution method [22]. Briefly, *C. neoformans* inoculums treated with different concentrations of Complex **1** for 24 h were taken and plated on drug-free yeast extract–peptone–dextrose (YPD) agar and incubated for another 2 days to measure colony-forming units (CFUs). The MFC was considered the lowest concentration of Complex **1** that allowed less than 0.1% of the original inoculum treated with Complex **1** to grow on the surface of the agar. As displayed in Figure 2, the results showed that the number of CFU were consistent with decreasing amounts of drug and the MFC of *C. neoformans* grown with Complex **1** was 11.3 μM.

### 2.4. A Comparative Time-Course Assay of Cell Viability

**Figure 3:**
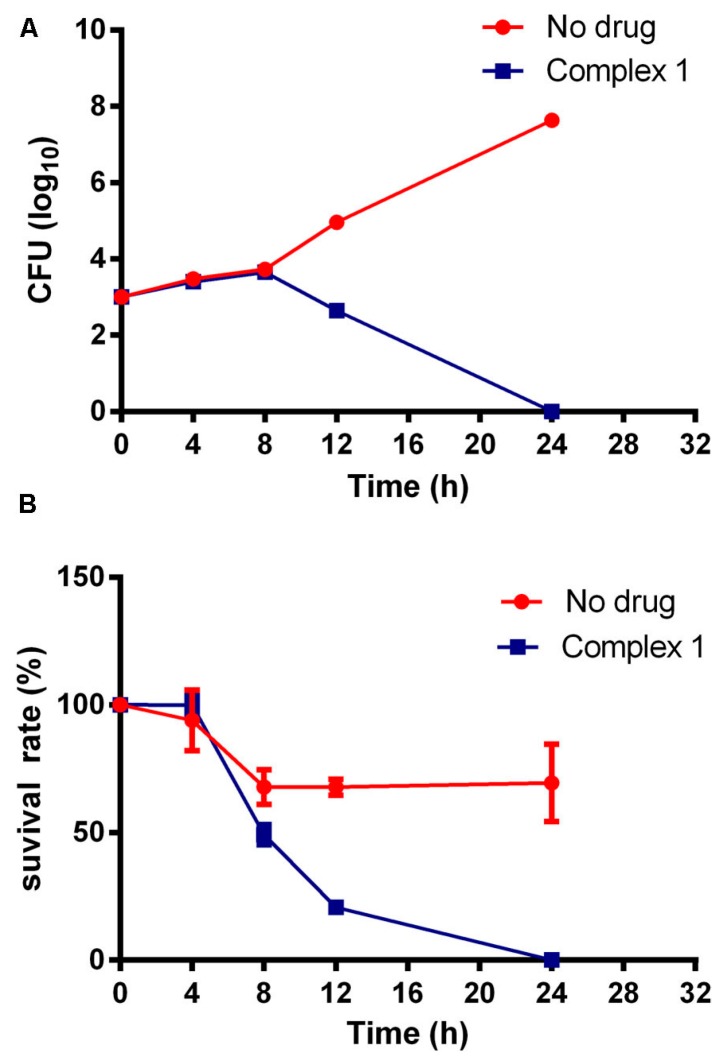
Complex **1** is fungicidal against both proliferative and quiescent Cryptococcus cells. (**A**) H99 cells were inoculated into RPMI1640 and (**B**) into PBS, and cultured without any drug (control) or in the presence of Complex **1** (17.92 μM). At the indicated time points, aliquots of cell suspensions were transferred and plated on the drug-free yeast extract–peptone–dextrose (YPD) medium to determine CFUs after 48 more hours of incubation. Fungal cells proliferated rapidly in the absence of any drugs, but they were gradually killed with Complex **1** treatment.

It is commonly known that drugs that can kill fungal pathogens lead to a quick reduction of fungal burden and the restriction of the prevalence of drug-resistant strains [23,24,25]. To investigate whether Complex **1** is fungistatic or fungicidal, a comparative time-course assay of cell viability was performed. We cultured *C. neoformans* H99 cells in an RPMI1640 medium or in a PBS buffer to represent rapidly proliferative or quiescent cells respectively. These cells were then treated with no drug or with Complex **1**. At different time points, aliquots of cell suspensions were spread onto the drug-free YPD medium to determine the number of viable cells by measuring the CFUs. Figure 3 reveals that the number of viable cryptococcal cells began to decrease at 12 h in the presence of Complex **1** in RPMI, which was contrary to the no-drug control, and there were no viable cells left after 24 h of incubation. Similarly, H99 cells in PBS were cleared by Complex **1** after 24 h of incubation. These results demonstrated that Complex **1** was able to kill fungal cells independently regardless of what growth stage the cells were in.

### 2.5. Antifungal Mechanism

**Figure 4:**
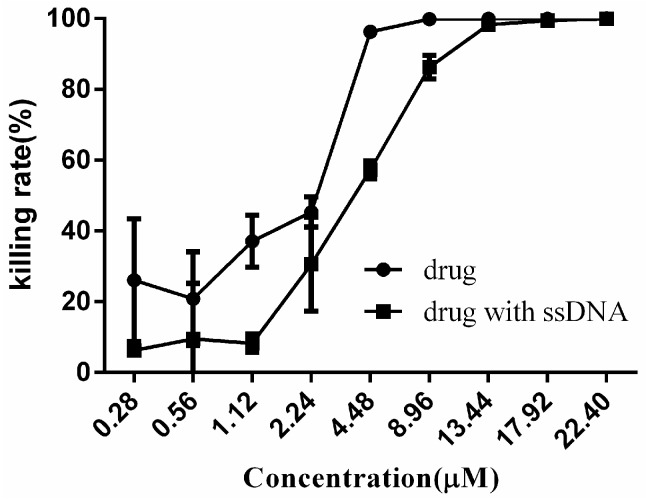
Killing rate of Complex **1** in the absence and presence of exogenous DNA. ssDNA with a concentration of 100 μg/mL was incubated with different concentrations of Complex **1** for 30 min and added to *C. neoformans* culture for treatment for about 24 h. The number of viable cells was determined by plating the suspension on drug-free solid media and then measuring CFUs.

Finally, we preliminarily investigated the antifungal mechanism of Complex **1**. Since Complex **1** did exhibit a strong stabilization of DNA duplex in vitro [11], we speculated that genomic DNA of *C. neoformans* might also be a potential target of Complex **1**. Therefore, we tested the effects of Complex **1** on the growth of *C. neofromans* in the absence and presence of exogenous salmon sperm DNA (ssDNA). As shown in Figure 4, when the concentration of Complex **1** ranged from 0.28 to 8.96 μM, it did significantly affect the antimicrobial activity because of the existence of exogenous DNA. Apparently, exogenous DNA could weaken the efficacy of Complex **1** and rescue *C. neoformans* from growth inhibition to a certain extent. The result also showed that the dosage of Complex **1** with exogenous DNA had to be increased from 8.96 to 13.44 μM to achieve a 100% killing rate, compared to the control group, suggesting the antifungal process may be relevant with DNA binding.

## 3. Materials and Methods

All reactions were carried out under an argon atmosphere. Chloroform was distilled under nitrogen from calcium hydride. 1,10-Phenanthroline was sublimated before use. Ampicillin and fluconazole were brought from Meilun Biotech Co. (Dalian, China), morpholinepropanesulfonic acid, and Salmon Sperm DNA were obtained from Sigma-Aldrich (St. Louis, MO, USA). Their stock solutions were freshly prepared, filter-sterilized, and used at indicated concentrations. SiI_4_ was purchased from Alfa, and the other reagents were purchased from Sigma, Acros, or Strem and used without further purification. Column chromatography was performed with silica gel (230–400 mesh). ^1^H- and ^13^C-NMR spectra were recorded on a Bruker Advance (400 MHz, Bruker Corporatio, Billerica, MA, USA) at ambient temperature.

In total, the following bacterial and fungal strains were used for this study: *Cryptococcus neoformans* H99, *Candida albicans* ATCC90028, *Staphylococcus aureus* ATCC 33591(MRSA), ATCC 25923(MSSA), *Escherichia coli* ATCC25922, *Pseudomonas aeruginosa* PAO1, and clinical isolated *Acetobacter baumannii*. *S. aureus*, *E. coli*, and *P. aeruginosa* were grown in TSB medium; *C. neoformans* and *C. albicans* were grown in YPD medium. These strains were stored as glycerin stock in −80 °C.

### 3.1. Syntheses

**Figure 5:**
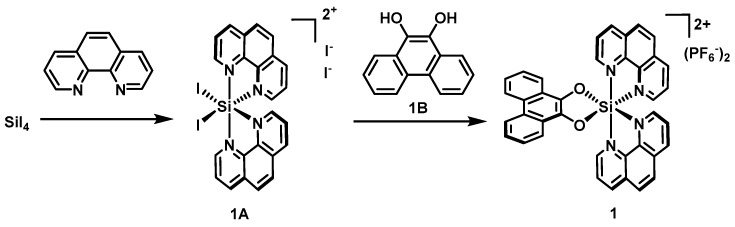
Synthetic route of Complex **1**.

The silicon complex **1** was synthesized by modified literature procedures (Figure 5) [11]. Accordingly, a suspension of silicon(IV) iodide (2.0 g, 3.74 mmol) and 1,10-phenanthroline (1.5 g, 8.37 mmol) in freshly distilled CHCl_3_ (50 mL) was purged with Argon for 15min and was heated to 55 °C for 72 h under argon. The resulting brownish red suspension was cooled to room temperature and filtered to remove the solvent. The solid was washed with dry CHCl_3_ (2 × 25 mL), MeOH (25 mL), and Et_2_O (25 mL) successively and was subsequently dried in vacuum to afford the deep-brownish solid **1A** (2.6 g, 77%), which could be used directly for the next step without further purification. In addition, phenanthrene-9,10-diol (Compound **1B**) was prepared exactly according to the published procedures [26].

A suspension of **1A** (200 mg, 0.22 mmol) and 4 equivalents of **1B** (185 mg, 0.88 mmol) in freshly distilled CHCl_3_ (30 mL) was purged with argon for 15 min and then stirred at 55 °C for 3 h. The resulting dark-brown suspension was cooled to room temperature, and the solvent was removed in vacuo. The residue was dissolved in 5 mL of mixed solvent {CH_3_CN:H_2_O:KNO_3_(sat.) = 100:3:1} and subjected to silica gel chromatography with CH_3_CN first and afterward switched to CH_3_CN:H_2_O:KNO_3_(sat) = 200:3:1 and CH_3_CN:H_2_O:KNO_3_(sat) = 100:3:1. The product eluents were concentrated and redissolved in 10 mL of water, and the product was precipitated by the addition of excessive solid NH_4_PF_6_. The precipitate was centrifuged, washed twice with water (2 × 10 mL), and dried to afford the pure diolate complex **1** as a pale-brown solid (52 mg, 27%). The NMR data of Complex **1** were consistent withthe reported data [11].

The ruthenium complexes **2** and **3** [27] and phenanthrene-9,10-diol [28] (**1B**) were prepared according to the published procedures.

### 3.2. Disk Diffusion Susceptibility Test

The disk diffusion method was used to determine sensitivity of bacteria and fungi to Complexes **1**–**3** [21]. Bacteria and fungi direct colony suspensions were prepared in TSB and YPD from overnight culture, respectively. The turbidity of five different bacterial and two fungal suspensions were standardized to match 1.0 × 10^8^ CFU/mL. A sterile swab dipped in fresh bacterial or fungal suspensions were inoculated to the entire surface of TSB or YPD agar and inoculum was distributed evenly. Five microliters of complexes **1**–**3** (25 μg), ampicillin (25 μg), and fluconazole (25 μg) were impregnated on sterile paper disks for 15 min. Subsequently, the paper disks were aseptically placed on and pressed down firmly to the inoculated plates. Plates were incubated for 24 h for bacteria and *C. albicans*, and 48 h for *C. neoformans* in a 37 °C incubator. After incubation, the inhibition zones were measured. The presence or absence of growth around the disks was a measure of the ability of complexes **1**–**3** to inhibit the growth of bacteria and fungi. All experiments were done in triplicate.

### 3.3. Determination of MIC and MFC Values

The MIC values were determined using the broth microdilution methods developed by the Clinical and Laboratory Standards Institute (CLSI) and published in document M27-A3 [22]. RPMI1640 with 0.165 M morpholinepropanesulfonic acid (MOPS) (pH 7.0) was used as the test medium.

The final concentrations of antifungal agents studied ranged from 0.14 to 71.68 μM for Complex **1**. The cell suspensions were diluted with the test medium to obtain an inoculum size of 1 × 10^3^ CFU/mL for *C. neoformans* H99. MICs were determined after incubation at 37 °C for 48 h. The MIC endpoints were defined as the lowest drug concentration that leads to a reduction in growth of 50% or more compared with the growth. The MFC was considered as the lowest concentration of Complex **1** that allowed less than 0.1% of the original inoculum.

### 3.4. Anti-Fungal Activity of Complex ***1*** on *C. neoformans* in the Presence of ssDNA

Salmon Sperm DNA (ssDNA) (Sigma-Aldrich) with a concentration of 100 μg/mL was incubated with different concentrations of Complex **1** for 30 min in a 37 °C incubator. Then, the mixture was added to the *C. neoformans* culture and incubated at 37 °C for 24 h in tubes with constant shaking. The number of viable cells was determined by plating the suspension on drug-free solid media and then measuring CFUs.

## 4. Conclusions

In conclusion, we here reported for the first time that the octahedral silicon complex **1** can very well serve as an efficient antimicrobial agent. The silicon complex not only significantly inhibited the growth of *S. aureus* but also displayed selective antifungal activity against *C. neoformans*. Although the exact antimicrobial mechanism of this silicon complex needs to be further investigated, we are convinced that this work will pave the way to the development of novel hexacoordinate silicon-based antifungal drugs. Work along these lines as well as the biological applications of other silicon complexes with analogous structures are in progress.

## Figures and Tables

**Table 1:** In vitro anti-fungal activity towards *C. neoformans* of Complexes **1**–**3** and typical positive control drugs (MIC, μM).

Drugs	*C. neoformans*
**1**	4.5
**2**	34.3
**3**	74.4
fluconazole	6.5
amphotericin B	0.3
5-flucytosine	3.9

## Abbreviations

YPD: yeast extract–peptone–dextrose; CFU: colony-forming unit; PBS: phosphate-buffered saline; MOPS: morpholinepropanesulfonic acid; ssDNA: Salmon Sperm DNA.
